# Cognitive and Neuropsychiatric Function in Former American Football Players

**DOI:** 10.1001/jamanetworkopen.2025.60077

**Published:** 2026-02-27

**Authors:** Anna Aaronson, Grace Badlam, Shania C. Mulayi, Fatima Tuz-Zahra, Kelsey J. Goostrey, Yorghos Tripodis, William S. Cole-French, Matthew Roebuck, Greta Schneider, Brittany N. Pine, Joseph N. Palmisano, Brett M. Martin, Kenton H. Zavitz, Douglas I. Katz, Christopher J. Nowinski, Ann C. McKee, Thor D. Stein, R. Scott Mackin, Michael D. McClean, Jennifer Weuve, Jesse Mez, Michael W. Weiner, Rachel L. Nosheny, Robert A. Stern, Michael L. Alosco

**Affiliations:** 1 Boston University Alzheimer’s Disease Research Center, Boston University CTE Center, Boston University Chobanian & Avedisian School of Medicine, Boston, Massachusetts; 2 Department of Biostatistics, Boston University School of Public Health, Boston, Massachusetts; 3 Center for Health Data Science, Boston University School of Public Health, Boston, Massachusetts; 4 Cambridge Cognition, Cambridge, Massachusetts; 5 Department of Neurology, Boston University Chobanian & Avedisian School of Medicine Boston, Massachusetts; 6 Department of Neurology, Boston Medical Center, Boston, Massachusetts; 7 Concussion Legacy Foundation, Boston, Massachusetts; 8 US Department of Veterans Affairs (VA) Bedford Healthcare System, Bedford, Massachusetts; 9 VA Boston Healthcare System Jamaica Plain, Massachusetts; 10 Department of Pathology and Laboratory Medicine, Boston University Chobanian & Avedisian School of Medicine, Boston, Massachusetts; 11 VA Advanced Research Center, San Francisco Veteran’s Administration Medical Center, San Francisco, California; 12 Department of Psychiatry and Behavioral Sciences, University of California, San Francisco; 13 Department of Epidemiology, Boston University School of Public Health, Boston, Massachusetts; 14 Department of Radiology and Biomedical Imaging, University of California, San Francisco; 15 Department of Neurology, University of California, San Francisco; 16 Department of Medicine, University of California, San Francisco; 17 Northern California Institute for Research and Education, San Francisco; 18 Department of Neurosurgery, Boston University Chobanian & Avedisian School of Medicine, Boston, Massachusetts; 19 Department of Anatomy and Neurobiology, Boston University Chobanian & Avedisian School of Medicine, Boston, Massachusetts

## Abstract

**Importance:**

Repetitive head impacts (RHI) from American football have been associated with later-life cognitive and neuropsychiatric changes. Findings have been limited by small samples, focus on elite players, and lack of appropriate control groups.

**Objective:**

To assess the association between American football participation and cognitive and neuropsychiatric function in men 40 years or older.

**Design, Setting, and Participants:**

In this cross-sectional study, online data from male football players in the Head Impact & Trauma Surveillance Study (HITSS) were collected between March 7, 2022, and April 9, 2025. In a subanalysis, football players were matched to Brain Health Registry controls without RHI.

**Exposures:**

RHI proxies, including self-reported total years of football play, highest level of play, position, and age of first exposure.

**Main Outcomes and Measures:**

Linear regression models with multiway cluster-robust SEs compared performance of players vs controls on computerized cognitive tests (Cambridge Automated Neuropsychological Battery Paired Associates Learning Test First Attempt Memory Score [PALFAMS] and Total Errors Adjusted [PALTEA]), subjective cognitive concerns (Everyday Cognition Scale [ECog]), and depressive symptoms (Geriatric Depression Scale 15 [GDS-15]). Multivariable linear regressions, analyses of covariance, and binary logistic regression models tested associations between RHI proxies and PALFAMS, PALTEA, ECog, GDS-15, and additional neuropsychiatric (Behavior Rating Inventory of Executive Function–Adult [BRIEF-A] Behavioral Regulation Index [BRI]) and cognitive (BRIEF-A Meta-Cognition Index [MI]) measures. Models were adjusted for age, race, educational level, and vascular risk.

**Results:**

The study sample included 3970 male former American football players (mean [SD] age, 55.93 [10.00] years) enrolled in HITSS. Two substudies were performed: (1) all 3970 football players and (2) 943 players and controls (mean [SD] age, 58.46 [10.37] years), including 661 football players and 282 Brain Health Registry controls. Overall, all study participants were highly educated (561 [85%] had a ≥4-year degree). Compared with controls, players had worse scores on PALFAMS (B = −0.64; 95% CI, −1.23 to 0.05; β = −0.15; *P* = .03), PALTEA (B = 0.31; 95% CI, 0.07-0.54; β = 0.18; *P* = .01), ECog (B = 0.11; 95% CI, 0.07-0.15; β = 0.38; *P* < .001), and GDS-15 (B = 0.62; 95% CI, 0.39-0.86; β = 0.37; *P* < .001). Among the 3970 total players (mean [SD] years of education, 16.11 [2.20]), professional players had worse scores than college and high school or youth players. Years of play was associated with higher ECog (B = 0.006; 95% CI, 0.003-0.009; adjusted *P* < .001), MI (B = 0.17; 95% CI, 0.02-0.32; adjusted *P* = .04), BRI (B = 0.27; 95% CI, 0.16-0.37; adjusted *P* < .001), GDS-15 (B = 0.03; 95% CI, 0.009-0.04; adjusted *P* = .003), and PALTEA (B = 0.03; 95% CI, 0.004-0.06; adjusted *P* = .04) scores.

**Conclusions and Relevance:**

In this cross-sectional study of former American football players, prior American football participation was associated with worse later-life cognitive and neuropsychiatric function. These findings support a dose-response association with years and level of play, providing context to help clinicians and researchers assess the risk of symptoms among former players.

## Key Points

**Question:**

What is the association between prior American football participation and cognitive and neuropsychiatric function in men 40 years or older?

**Findings:**

In this cross-sectional study of 3970 former football players, higher level of football play and more years of football play were associated with worse computerized cognitive test performance and greater neuropsychiatric symptoms. Compared with 282 matched controls, 661 former football players performed worse on a computerized cognitive test, had more subjective cognitive concerns, and had more severe depressive symptoms.

**Meaning:**

These findings support an association between football play and worse later-life cognitive and neuropsychiatric function.

## Teaser

This cross-sectional study of American football players examines whether prior football participation is associated with worse cognitive and neuropsychiatric function at age 40 years or older.

## Introduction

Repetitive head impacts (RHI) incurred through American football play can result in symptomatic and asymptomatic traumatic brain injuries (TBI).^1,2^ Autopsy studies demonstrate that cumulative RHI exposure from American football can confer risk of the neurodegenerative disease chronic traumatic encephalopathy,^3,4,5,6^ alongside related neurodegenerative conditions^7,8,9,10,11^ and other neuropathological conditions (eg, white matter degeneration).^12^ In vivo neuroimaging studies of living former American football players similarly show evidence of structural,^13,14,15,16^ functional,^17,18^ and molecular^19,20^ brain changes.

Studies^21,22,23,24,25,26,27^ have found associations between higher football exposure, as indexed by proxies (eg, age of first exposure and total years of play), and later-life cognitive and neuropsychiatric function (eg, depression, apathy, and neurobehavioral changes). Literature^5,28,29,30,31^ is inconsistent on the presence, type, and severity of clinical symptoms, in addition to uncertainty regarding their association with RHI. These inconsistencies could partially stem from methodological differences, including varied definitions of RHI exposure.^25^ Exposed groups in most studies^32,33,34^ have consisted of small samples of elite football players at the exclusion of individuals who played at high school and youth levels. Use of adequately comparable, non–football-playing control groups has also been uncommon. Long-term cognitive and neuropsychiatric effects of RHI exposure from American football remain poorly understood, particularly in terms of how observed associations generalize to all football players.

We examined cross-sectional association of prior American football participation across all levels of play (youth, high school, college, and professional) with cognitive and neuropsychiatric function, leveraging a cohort of former American football players 40 years or older. Two substudies comprised this investigation. In the first, we evaluated differences in cognitive and neuropsychiatric outcomes between a subset of the former American football players and a demographically well-matched group of men not exposed to RHI. In the second substudy, we used data solely from the football player cohort to evaluate RHI exposure proxies (years of play, age of first exposure, highest level of play, and position) associated with outcomes. We hypothesized that the football cohort would perform worse on cognitive and neuropsychiatric measures compared with the controls and that greater RHI exposure from football play would be associated with worse cognitive function, greater subjective cognitive concerns, and greater depressive symptoms.

## Methods

### Study Design

The sample included male former American football players enrolled in the Head Impact & Trauma Surveillance Study (HITSS). HITSS is a longitudinal, observational, online study of former American football and soccer players 40 years or older from the US. HITSS is an extension of the University of California, San Francisco Brain Health Registry (BHR), an online dementia research registry for the longitudinal monitoring of more than 100 000 adults.^35,36^ HITSS participants were recruited via separate channels than BHR participants (eMethods 1 in Supplement 1). HITSS aims to characterize risks of later-life brain health concerns among former American football and soccer players across all levels of play. HITSS participants complete BHR assessments, additional modules measuring RHI and TBI exposure, and cognitive and neuropsychiatric assessments. The 90-minute HITSS battery includes self-report questionnaires pertaining to demographics, sports participation, TBI and medical history, health behaviors, neuropsychiatric and neurobehavioral symptoms, and computerized cognitive tests. Participants are not compensated for task completion but are entered into a drawing for the opportunity to win a $500 gift card. HITSS participants must be 40 years or older, have played organized soccer or American football at any level, and have computer access. This article focuses on American football. Participants provided written informed consent. HITSS is approved by the Boston University Medical Campus Institutional Review Board. This study includes data collected from participants’ baseline visits between March 7, 2022, and April 9, 2025. This study adheres to the Strengthening the Reporting of Observational Studies in Epidemiology (STROBE) reporting guidelines.

### Samples

**Figure 1.**
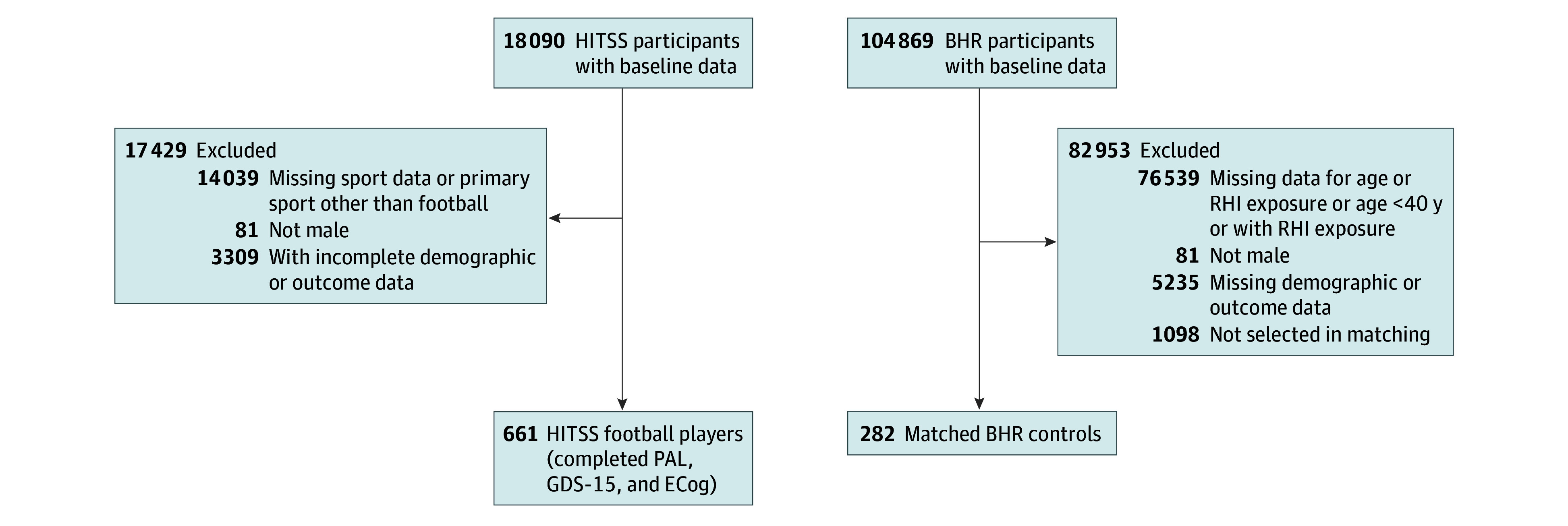
Participant Flowchart and Sample Derivation Derivation of the sample of American football players from the Head Impact & Trauma Surveillance Study (HITSS) and propensity score matched–controls without repetitive head impact (RHI) exposure. BHR indicates Brain Health Registry; ECog, Everyday Cognition Scale; GDS-15, Geriatric Depression Scale-15; PAL, Paired Associates Learning.

The first substudy assessed HITSS participants who played football and BHR participants who denied RHI (Figure 1). Participants were included in the football cohort if they self-identified as male, endorsed football as their primary sport, and indicated that they were no longer playing football. Many athletes play multiple sports, so to reflect the population of former football players, we did not exclude individuals who played other contact sports (eg, soccer and ice hockey) or military service (eTable 1 in Supplement 1). Controls were male BHR participants 40 years or older who answered no to the following question: “Have you ever had a period of time in which you experienced repeated impacts to your head (eg, history of abuse, contact sports, military duty)?” on an online adaptation of the Ohio State University TBI Identification Method.^37^ Controls were matched to a subset of football players who completed the Geriatric Depression Scale 15 (GDS-15), Everyday Cognition Scale (ECog), and Cambridge Automated Neuropsychological Battery Paired Associates Learning Test First Attempt Memory Score (PALFAMS) and Total Errors Adjusted (PALTEA), without missing demographic data based on propensity score. The propensity score was based on age, race (eMethods 4 in Supplement 1), ethnicity, and educational level. Race categories included African American or Black, Asian, Native American, White, multiple races, and other race (HITSS category); and ethnicity categories included Latino or not Latino. Race and ethnicity data were collected because these factors are associated with dementia risk and incidence.^38,39^ Matching is described in eMethods 5 and eFigure 1 in Supplement 1. The second substudy evaluated associations between RHI proxies and cognitive and neuropsychiatric outcomes among HITSS former football players only.

### Measures

HITSS participants completed the Boston University RHI Exposure Assessment, which evaluates sports participation and other RHI sources (eMethods 2 in Supplement 1).^40^ We examined 4 measures of football exposure: (1) age of first exposure (AFE) to football (the age at which the participant began playing organized American football), (2) total years of football play (calculated by subtracting the AFE from the age at which the participant stopped playing football), (3) position of play (lineman or nonlineman), and (4) highest level of play (professional, college, high school, or youth). Professional level of play consisted of National Football League (NFL), Canadian Football League, Arena Football League, NFL Europe, and XFL. No semiprofessional leagues were included. Youth and high school were combined into one category due to small sample size of youth-only players (n = 97).

### Outcomes

HITSS includes remote, unsupervised, computerized cognitive tests and self-report questionnaires that assess domains affected by RHI, including executive function and episodic memory.^22,41,42,43,44^ Only tests common to both the HITSS and BHR batteries (PALFAMS, PALTEA, GDS-15, and ECog) were examined in the analyses of players and nonplayers. Analyses of the larger football cohort included the Behavior Rating Inventory of Executive Function–Adult (BRIEF-A) Meta-Cognition Index (MI) and Behavioral Regulation Index (BRI) (eMethods 3 in Supplement 1).

### Statistical Analysis

#### Football vs No RHI 

Analyses were performed using R, version 4.3.3 (R Foundation for Statistical Computing). We quantified associations of football-playing history (vs no RHI exposure) with each of the 4 outcomes assessed across HITSS and BHR (PALFAMS, PALTEA, ECog, and GDS-15), using mean differences between the groups in each outcome. We estimated these mean differences by fitting a separate regression model for each outcome. To account for the dependency structure introduced by the matching procedure and the reuse of controls, we used multiway cluster-robust SEs clustered by both participant identification and matched pair membership.^45^ We used a doubly robust estimation approach by including age as a covariate in all models to account for residual imbalance after matching. Standardized β values were reported as the measure of effect size.

#### RHI Proxies in the Football Cohort 

We characterized associations of the 4 RHI proxies among former football players with each of the 6 outcomes assessed in HITSS, using several approaches. We fitted a multiple linear regression model for each RHI measure-outcome pair. Models were parameterized to estimate the mean difference in each outcome: per each year older in AFE, per each additional year of play, and among linemen vs nonlinemen.

Analysis of covariance (ANCOVA) compared outcomes across each pair of levels of play. Omnibus tests assessed the overall effect of highest level of play; partial η^2^ was reported as the measure of effect size. We estimated marginal means, including SEs and 95% CIs, across each level of play (eTable 2 in Supplement 1). Post hoc Tukey-adjusted pairwise comparisons examined differences in performance. Sample sizes varied across outcomes due to missing data (Table 1).

**Table 1.** Characteristics of the American Football and Control Groups

Characteristic	No. (%) of participants^a^		
	Overall (N = 943)	Control group (n = 282)	Football group (n = 661)
Age, mean (SD), y^b^	58.46 (10.37)	60.44 (9.71)	57.62 (10.53)
Educational level^b^			
High school	10 (1.1)	2 (0.7)	8 (1.2)
Some college	78 (8.2)	22 (7.7)	56 (8.5)
2-y Degree	52 (5.5)	17 (5.9)	35 (5.3)
4-y Degree	340 (36)	93 (33.0)	247 (37.0)
Master’s degree	317 (34.0)	89 (32.0)	228 (34.0)
Doctoral degree	66 (6.9)	23 (7.7)	43 (6.5)
Professional degree	78 (8.3)	35 (12.0)	43 (6.5)
Prefer not to say	2 (0.2)	1 (0.3)	1 (0.2)
Race^b^			
African American or Black	42 (4.5)	12 (4.3)	30 (4.5)
Asian	7 (0.7)	3 (1.1)	4 (0.6)
Native American	4 (0.4)	0	4 (0.6)
White	858 (91.0)	258 (91.0)	600 (91.0)
Multiple races	28 (3.0)	8 (2.8)	20 (3.0)
Other race^c^	4 (0.4)	1 (0.4)	3 (0.5)
Ethnicity^b^			
Latino	32 (3.4)	8 (2.8)	24 (3.6)
Not Latino	911 (97.0)	274 (97.0)	637 (96.0)
Outcome measures, mean (SD) score			
PALFAMS	12.26 (4.23)	12.41 (4.32)	12.20 (4.20)
PALTEA	16.44 (14.06)	16.06 (14.48)	16.61 (13.88)
ECog	1.51 (0.53)	1.38 (0.43)	1.56 (0.56)
GDS-15	6.02 (1.71)	5.56 (1.48)	6.22 (1.77)
Vascular risk^d^	608 (65.8)	177 (62.8)	431 (65.2)

Abbreviations: ECog, Everyday Cognition Scale; GDS-15, Geriatric Depression Scale-15; PALFAMS, Paired Associates Learning First Attempt Memory Score; PALTEA, Paired Associates Learning Total Errors Adjusted.

^a^ Unless otherwise indicated.

^b^ These variables were used in the propensity score matching process.

^c^ Head Impact & Trauma Surveillance Study category.

^d^ Due to missing data, only 924 participants had vascular risk measures available.

Linear regression and ANCOVA model *P* values were adjusted by applying the Benjamini-Hochberg false discovery rate method independently for each RHI proxy. The total number of hypotheses was set to 4 for the cognitive domain (PALFAMS, PALTEA, MI, and ECog) and 2 for the neuropsychiatric domain (GDS-15 and BRI). An FDR-adjusted, 2-sided *P* ≤ .05 was considered statistically significant. ECog was log and PALTEA was square root transformed in linear regressions and ANCOVA due to nonnormal distribution of residuals.

To investigate clinical relevance of significant group effects observed in the linear models and ANCOVA, we conducted binary logistic regressions using impairment cut points for the BRIEF-A MI and BRI (T-score, ≥65), ECog (T-score, ≥1.31^46,47^), and GDS-15 (cut point, ≥5).^48^ These analyses examined whether the RHI proxies were associated with increased odds of clinically meaningful elevations on these outcomes. All linear and logistic regression and ANCOVA models adjusted for age, educational level, race, and vascular risk (participants classified as being at vascular risk if they endorsed having any of the following: heart disease, high blood pressure, high cholesterol, or diabetes).

#### Sensitivity Analyses

Four additional linear regression models with multiway cluster-robust SEs compared performance of controls with each level of football player on all outcomes, adjusting for age. The number of participants for whom youth was the highest level of play among the football cohort was small (n = 97). Therefore, we also estimated the associations of highest level of play with the outcomes excluding youth players. We conducted all linear regressions and ANCOVAs adjusting for self-report history of alcohol and drug abuse as covariates.

## Results

The study sample included 3970 male former American football players (mean [SD] age, 55.93 [10.00] years; 471 [12.0%] African American or Black, 16 [0.4%] Asian, 30 [0.8%] Native American, 20 [0.5%] Pacific Islander, 3198 [81.0%] White, 122 [3.1%] multiple races, and 67 [1.7%] other race) enrolled in HITSS. Two substudies were performed: (1) all 3970 football players and (2) 943 players and controls (mean [SD] age, 58.46 [10.37] years; 42 [4.5%] African American or Black, 7 [0.7%] Asian, 4 [0.4%] Native American, 858 [91.0%] White, 8 [2.8%] multiple races, and 4 [0.4%] other race), including 661 football players and 282 Brain Health Registry controls. Table 1 lists the characteristics of the football and control groups. Table 2 lists the characteristics of all the football players by highest level of play. eTable 3 stratifies Table 1 by level of play. Linear regression models with multiway cluster-robust SEs revealed that, compared with controls, football players had worse scores on PALFAMS (B = −0.64; 95% CI, −1.23 to 0.05; β = −0.15; *P* = .03), square root PALTEA (B = 0.31; 95% CI, 0.07-0.54; β = 0.18; *P* = .01), log ECog (B = 0.11; 95% CI, 0.07-0.15; β = 0.38; *P* < .001), and GDS-15 (B = 0.62; 95% CI, 0.39-0.86; β = 0.37; *P* < .001). In the cohort of 3970 football players, those who completed PAL were older, had more education, and had a lower proportion of professional players (eTable 4 in Supplement).

**Table 2.** American Football Player Characteristics by Highest Level of Play

Characteristic	No. (%) of participants^a^				*P* value^b^
	Overall (N = 3970)	Youth or high school (n = 1833)	College (n = 1542)	Professional (n = 595)	
Age, mean (SD), y	55.93 (10.00)	55.74 (9.81)	56.27 (10.12)	55.64 (10.29)	.28
Education					
Length of education, mean (SD), y	16.11 (2.20)	15.79 (2.34)	16.73 (1.88)	15.50 (2.12)	<.001
Missing	9	4	2	3	NA
Race (n = 3924)					
African American or Black	471 (12.0)	154 (8.5)	155 (10.0)	162 (28.0)	<.001
Asian	16 (0.4)	10 (0.6)	4 (0.3)	2 (0.3)	
Native American	30 (0.8)	14 (0.8)	11 (0.7)	5 (0.9)	
Pacific Islander	20 (0.5)	3 (0.2)	9 (0.6)	8 (1.4)	
White	3198 (81.0)	1534 (85)	1300 (85.0)	364 (62.0)	
Multiple races	122 (3.1)	58 (3.2)	37 (2.4)	27 (4.6)	
Other race	67 (1.7)	38 (2.1)	11 (0.7)	18 (3.1)	
Missing	46	22	15	9	
Ethnicity (n = 3867)					
Latino	165 (4.3)	90 (5.1)	36 (2.4)	39 (6.8)	<.001
Not Latino	3702 (96)	1691 (95)	1476 (98)	535 (93.0)	
Missing	103	52	30	21	NA
Vascular risk (n = 2923)					
Yes	1974 (68.0)	965 (69.0)	754 (66.0)	255 (66.0)	.21
Missing	1047	437	404	206	
American football play					
Duration of football play, mean (SD), y (n = 3944)	8.75 (4.61)	6.24 (2.89)	9.79 (3.07)	13.88 (6.63)	<.001
Missing	26	12	1	13	NA
American football exposure					
AFE to football, mean (SD), y (n = 3950)	10.73 (2.73)	10.91 (2.65)	10.63 (2.56)	10.44 (3.32)	<.001
Missing	20	9	8	3	NA
Linemen (n = 3878)					
Yes	1651 (43.0)	783 (43.0)	670 (44.0)	198 (38.0)	.054
Missing	92	13	8	71	
Cognitive measure scores					
ECog, mean (SD) (n = 2682)	1.59 (0.57)	1.56 (0.54)	1.60 (0.57)	1.70 (0.65)	.004
Impaired	1573 (59.0)	740 (57.0)	616 (60.0)	217 (63.0)	.09
Missing	529	510	249	1288	NA
PALFAMS, mean (SD)	12.16 (4.22)	12.60 (4.27)	11.96 (4.09)	11.05 (4.26)	.008
PALTEA, mean (SD)	16.64 (13.89)	14.99 (13.11)	17.66 (14.20)	19.87 (15.16)	.002
Missing	3185	1466	1207	512	NA
BRIEF-A MI score, mean (SD) (n = 2480)	64.25 (17.12)	64.09 (17.01)	63.76 (17.07)	66.37 (17.62)	.06
Impaired	750 (30.0)	353 (30.0)	287 (29.0)	110 (35.0)	.18
Missing	1490	647	565	278	NA
Neuropsychiatric measures					
GDS-15 score (n = 2154)	6.45 (1.89)	6.41 (1.93)	6.37 (1.77)	6.83 (2.04)	.006
Impaired	1926 (89.0)	917 (88.0)	758 (90.0)	251 (94.0)	.006
Missing	1816	786	702	328	NA
BRIEF-A BRI score (n = 2480)	47.39 (12.43)	47.04 (12.16)	47.08 (12.30)	49.64 (13.61)	.01
Impaired	658 (27.0)	295 (25.0)	256 (26.0)	107 (34%)	.006

Abbreviations: AFE, age of first exposure to football; BRI, Behavioral Regulation Index; ECog, Everyday Cognition Scale; BRIEF-A, Behavior Rating Inventory of Executive Function–Adult; GDS-15, Geriatric Depression Scale-15; MI, Meta-Cognition Index; PALFAMS, Paired Associates Learning First Attempt Memory Score; PALTEA, Paired Associates Learning Total Errors Adjusted.

^a^ Unless otherwise indicated.

^b^ Kruskal-Wallis rank sum test, Pearson χ^2^ test, or Fisher exact test.

### Level of Play

**Figure 2.**
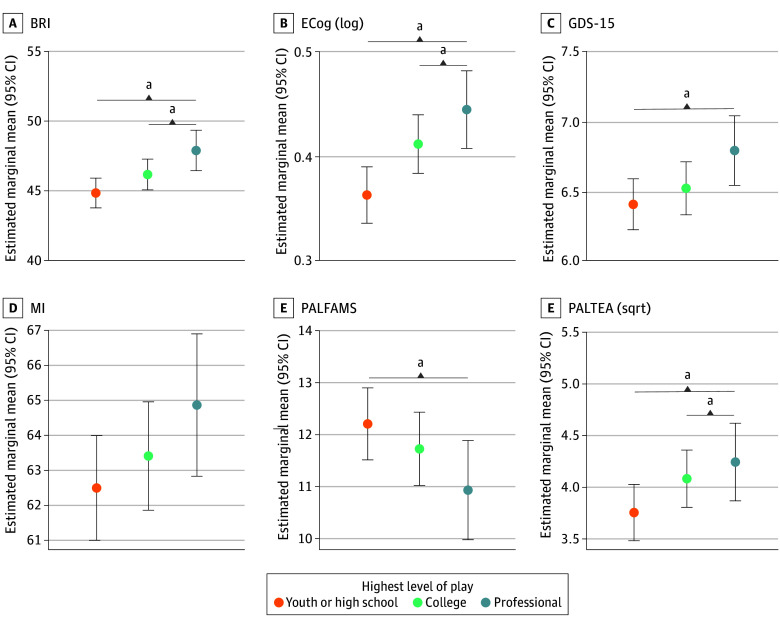
Cognitive and Neuropsychiatric Outcomes by Highest Level of American Football Play Plots depicting highest level of football play and estimated marginal means of outcome measures. Estimates are adjusted for age, educational level, race, and vascular risk. Everyday Cognition Scale (ECog) was log transformed in analyses, and Paired Associates Learning Total Errors Adjusted (PALTEA) was square root transformed due to nonnormal distribution of residuals. Higher ECog, Behavioral Regulation Index (BRI), and PALTEA scores indicate worse performance. GDS-15 indicates Geriatric Depression Scale-15; MI, Meta-Cognition Index; PALFAMS, Paired Associates Learning First Attempt Memory Score. ^a^Significant difference (*P* < .05).

ANCOVA models demonstrated a significant omnibus effect for highest level of play on PALFAMS (F = 3.88, adjusted *P* = .03, partial η^2^ = 0.02), square root PALTEA (F = 5.47, adjusted *P* = .009, partial η^2^ = 0.02), log ECog (F = 11.63, adjusted *P* < .001, partial η^2^ = 0.007), BRI (F = 8.95, adjusted *P* < .001, partial η^2^ = 0.005), and GDS-15 (F = 4.48, adjusted *P* = .01, partial η^2^ = 0.007). Post hoc Tukey-adjusted pairwise comparisons revealed professional players had worse scores compared with college and high school or youth, and college players had worse scores than high school or youth (Figure 2).

Binary logistic regression models demonstrated that, compared with youth or high school players, professionals had higher odds of clinically meaningful elevations on the following scales: ECog (odds ratio [OR], 1.36; 95% CI, 1.04-1.78; *P* = .02), BRI (OR, 1.61; 95% CI, 1.22-2.13; *P* < .001), and GDS-15 (OR, 2.21; 95% CI, 1.32-3.94; *P* = .004). College players had significantly higher odds of clinically meaningful elevations compared with youth or high school players on the following scales: ECog (OR, 1.29; 95% CI, 1.08-1.54; *P* = .006), BRI (OR, 1.29; 95% CI, 1.05-1.58; *P* = .01), and GDS-15 (OR, 1.42; 95% CI, 1.05-1.93; *P* = .02) (eTable 5 in Supplement 1).

### Years of Play

Multivariable linear regressions revealed significant associations between total years of play and higher BRI (B = 0.27; 95% CI, 0.16-0.37; adjusted *P* < .001), log ECog (B = 0.006; 95% CI, 0.003-0.009; adjusted *P* < .001), GDS-15 (B = 0.03; 95% CI, 0.009-0.04; adjusted *P* = .003), MI (B = 0.17; 95% CI, 0.02-0.32; adjusted *P* = .04), and square root PALTEA scores (B = 0.03; 95% CI, 0.004-0.06; adjusted *P* = .04) (Table 3). Binary logistic regression models demonstrated an association between more years of football play and higher odds of clinically meaningful elevation on BRI (OR, 1.04; 95% CI, 1.02-1.06; *P* < .001), ECog (OR, 1.03; 95% CI, 1.01-1.05; *P* = .003), and GDS-15 (OR, 1.07; 95% CI, 1.03-1.11; *P* < .001). There were no significant effects for AFE or position on any outcome.

**Table 3.** Associations Between Repetitive Head Impact Proxies and Cognitive and Neuropsychiatric Outcomes in the American Football Cohort^a^

Factor	B (95% CI) or F statistic	Adjusted *P* value
PALFAMS		
AFE	0.01 (−0.10 to 0.13)	.81
Years of play	−0.06 (−0.13 to 0.005)	.07
Linemen (yes)	0.10 (−0.45 to 0.65)	.72
Level of play	3.88	.03
PALTEA (square root)		
AFE	−0.008 (−0.05 to 0.04)	.81
Years of play	0.03 (0.004 to 0.06)	.04
Linemen (yes)	−0.04 (−0.26 to 0.18)	.72
Level of play	5.47	.009
ECog (log)		
AFE	−0.003 (−0.008 to 0.002)	.64
Years of play	0.006 (0.003 to 0.009)	<.001
Linemen (yes)	−0.02 (−0.05 to 0.004)	.42
Level of play	11.63	<.001
GDS-15		
AFE	0.01 (−0.02 to 0.05)	.40
Years of play	0.03 (0.009 0.04)	.003
Linemen (yes)	0.06 (−0.10 to 0.21)	.81
Level of play	4.48	.01
BRI		
AFE	−0.12 (−0.32 to 0.08)	.79
Years of play	0.27 (0.16 to 0.37)	<.001
Linemen (yes)	0.11 (−0.84 to 1.07)	.82
Level of play	8.95	<.001
MI		
AFE	−0.14 (−0.42 to 0.14)	.64
Years of play	0.17 (0.02 to 0.32)	.04
Linemen (yes)	0.60 (−0.74 to 1.94)	.72
Level of play	2.64	.07

Abbreviations: AFE, age of first exposure to football; BRI, Behavioral Regulation Index; ECog, Everyday Cognition Scale; GDS-15, Geriatric Depression Scale-15; MI, Meta-Cognition Index; PALFAMS, Paired Associates Learning First Attempt Memory Score; PALTEA, Paired Associates Learning Total Errors Adjusted.

^a^ Results from multivariable linear regression and analysis of variance models examining associations between repetitive head index proxies and cognitive and neuropsychiatric outcome measures. All models were adjusted for age, educational level, race, and vascular risk. *P* values were false discovery rate adjusted using the Benjamini-Hochberg method.

### Results of Sensitivity Analyses

Subgroup analyses substantiated a dose-response pattern across all outcomes, with effect sizes largest for professionals followed by the college and youth or high school levels, respectively (eTable 6 in Supplement 1). After removing youth players, there were no substantial changes in the direction or magnitude of effect sizes across models, and patterns of statistical significance remained consistent (eTable 7 and eFigure 2 in Supplement 1). The same was true after incorporating drug and alcohol abuse as covariates.

## Discussion

This study examined associations between football play and cognitive and neuropsychiatric outcomes in former American football players 40 years or older. Key findings included that (1) football players performed significantly worse on cognitive and neuropsychiatric outcomes compared with controls and (2) in the entire football cohort there were associations consistent with a dose-response pattern between years of play and highest level played and cognitive and neuropsychiatric measures. Findings demonstrate associations between multiple RHI proxies and later-life cognitive, mood, and behavioral impairments, thereby advancing the existing literature.^21,22,49,50,51,52^

Many previous studies examining cognitive and neuropsychiatric effects of football play failed to include players across all levels of play and well-matched control groups. When controls were included, sample sizes were small, and findings were mixed. One cohort study of men who played 1 season or more of high school football in the 1950s included matched controls and found no significant differences in later-life cognitive and depression-related outcomes between groups.^53^ Conversely, another study comparing neuropsychological and neuroimaging measures across 34 former NFL players and IQ-matched controls found higher rates of cognitive impairment and depression in the football players.^54^ Compared with controls, football players in our study performed worse on computerized cognitive tests and had more subjective cognitive concerns. They reported more depressive symptoms, aligning with previous findings tying football play to later-life depression, particularly in former elite players.^24,50,55,56^ A longitudinal study of US men found that individuals who reported 1 year or more of football play during adolescence did not display elevated depression risk and suicidality in their middle 30s to early 40s.^57^ The association between football play and depression is uncertain and may vary by age and level and duration of play.

Previous literature demonstrated a dose-response relationship between years of football play^58^ and level of play^59^ and chronic traumatic encephalopathy. However, associations between RHI and clinical outcomes have been inconsistent. This is likely due to heterogeneity in RHI operationalization, lack of validated tools for RHI quantification, limited range or variability due to focus on the upper end of exposure (ie, former professional players), selection biases in clinical and neuropathological brain bank studies, and lack of accounting for confounding individual risk factors (eg, genetics and medical comorbidities). Nonetheless, numerous studies have shown dose-response relationships between RHI exposure and clinical outcomes, including association of longer duration of football play with worse neurocognitive functioning in former NFL players^60^ and higher cumulative head impact exposure with later-life depression, apathy, and cognitive impairment in former high school and college players.^22^ Our study furnishes additional support for such associations.

We found no associations between AFE and our outcomes. The literature on AFE is mixed,^61^ and relationships between AFE and clinical symptoms have been found primarily in older symptomatic individuals. Among former professional and amateur players, Alosco et al^27^ identified an association between younger AFE to football and heightened odds for impairment in self-reported neuropsychiatric and executive function, whereas another study^62^ of 45 former NFL players failed to find an association between years of exposure to pre–high school football and neurological and neuropsychological outcomes. Studies have failed to find associations between AFE and neurocognitive outcomes in younger and healthier groups.^28,63^ A survey-based study also did not find an effect for AFE in middle-aged to older former adult high school football players,^29^ and neither did a study among 19 former NFL players.^30^ These inconsistencies could be partially attributable to lifespan effects. AFE may serve as an individual risk factor that may decrease resilience to pathology later in life rather than a direct causal factor. Our study did not find any significant associations between AFE and our outcomes, potentially due to our sample being younger (mean age, 55 years) and cognitively healthy (based on ECog).

### Limitations

This study has limitations. Selection biases limit generalizability of our findings, including requirement for an internet-connected device and digital literacy, lack of educational and ethnocultural diversity in the sample, and that individuals without mood and/or neurocognitive symptoms may be less likely to participate. This sample does not reflect the population of US football players.^64^ Only 785 football players completed a Paired Associates Learning Test, and this was not representative of the larger cohort. There are limitations to self-report data, including potential recall bias and error and missingness in some measures. There are also limitations to the way the propensity score matching procedure was conducted. Causality cannot be inferred by this study because matching was performed retrospectively rather than via random assignment to football vs nonfootball conditions. Additionally, there were likely geographic differences and differences in recruitment of HITSS vs BHR participants, which could have impacted study participation and motives for joining. Furthermore, unmeasured social determinants of health could affect outcomes independent of RHI. The analysis did not distinguish between starter and reserve players; because starters likely had highest intensity head impact exposure, this omission may bias the results toward the null. Additionally, sport history is not assessed in the BHR battery. Additionally, the small sample size of youth players was a limitation for ascertaining effects of youth play on outcomes.

## Conclusions

In this cross-sectional study of American football players from all levels of play and a well-matched control group, we observed a robust association consistent with a dose-response pattern between years and level of football play and cognitive and neuropsychiatric outcomes. Because millions of US men have played tackle football, understanding contributions of football exposure to brain health is crucial. Although the current results do not provide insights into individual risk, in combination with existing literature, consideration of years and level of football play offers a practical method to guide clinicians and researchers in determination of risk for later-life cognitive and neuropsychiatric symptoms. Future research should examine the role of individual risk and resilience factors (eg, genetics, medical comorbidities, and social determinants of health) on these outcomes.

## Notes

Supplement 1. **eMethods 1.** Participant recruitment into HITSS **eMethods 2.** Assessment of Football History **eMethods 3.** Description of Study Outcome Measures **eMethods 4.** Collection of Race and Ethnicity in HITSS and BHR **eMethods 5.** Detailed Description of and Rationale for Propensity Score Matching Procedure **eFigure 1.** Propensity Score Matching Diagnostics **eFigure 2.** Cognitive and Neuropsychiatric Outcomes by Highest Level of Football Play **eTable 1**. Sources of Potential RHI Exposure Besides Football **eTable 2.** Outcome Measure Estimated Marginal Means Across Highest Levels of Football Play **eTable 3.** Characteristics of Controls Versus Football Players (Stratified by Highest Level of Football Play) **eTable 4.** Characteristics of HITSS Participants Who Completed the Paired Associates Learning (PAL) Versus Those Who Did Not **eTable 5.** Associations Between RHI Proxies and Cognitive and Neuropsychiatric Outcomes in the Football Cohort (N=3,970) **eTable 6.** Subgroup Analyses Comparing Football Players to Controls **eTable 7.** Associations Between RHI Proxies and Cognitive/Neuropsychiatric Outcomes (Excluding Youth)
eReferences

Supplement 2.
Data Sharing Statement
