# Supplementary material for: Cognitive and Neuropsychiatric Function in Former American Football Players
Source: JAMA Netw Open. 2026 Feb 27;9(2):e2560077. doi: 10.1001/jamanetworkopen.2025.60077 (PMC12949441; doi:10.1001/jamanetworkopen.2025.60077)
Supplement: Supplement 1. — eMethods 1. Participant recruitment into HITSS eMethods 2. Assessment of Football History eMethods 3. Description of Study Outcome Measures eMethods 4. Collection of Race and Ethnicity in HITSS and BHR eMethods 5. Detailed Description of and Rationale for Propensity Score Matching Procedure eFigure 1. Propensity Score Matching Diagnostics eFigure 2. Cognitive and Neuropsychiatric Outcomes by Highest Level of Football Play eTable 1. Sources of Potential RHI Exposure Besides Football eTable 2. Outcome Measure Estimated Marginal Means Across Highest Levels of Football Play eTable 3. Characteristics of Controls Versus Football Players (Stratified by Highest Level of Football Play) eTable 4. Characteristics of HITSS Participants Who Completed the Paired Associates Learning (PAL) Versus Those Who Did Not eTable 5. Associations Between RHI Proxies and Cognitive and Neuropsychiatric Outcomes in the Football Cohort (N=3,970) eTable 6. Subgroup Analyses Comparing Football Players to Controls eTable 7. Associations Between RHI Proxies and Cognitive/Neuropsychiatric Outcomes (Excluding Youth) eReferences [file jamanetwopen-e2560077-s001.pdf]

Supplemental Online Content

Aaronson A, Badlam G, Mulayi SC, et al. Cognitive and neuropsychiatric function in former American football players. *JAMA Netw Open*. 2026;9(2):e2560077. doi:10.1001/jamanetworkopen.2025.60077

eMethods 1. Participant recruitment into HITSS
eMethods 2. Assessment of Football History
eMethods 3. Description of Study Outcome Measures
eMethods 4. Collection of Race and Ethnicity in HITSS and BHR
eMethods 5. Detailed Description of and Rationale for Propensity Score Matching Procedure
eFigure 1. Propensity Score Matching Diagnostics
eFigure 2. Cognitive and Neuropsychiatric Outcomes by Highest Level of Football Play
eTable 1. Sources of Potential RHI Exposure Besides Football
eTable 2. Outcome Measure Estimated Marginal Means Across Highest Levels of Football Play
eTable 3. Characteristics of Controls Versus Football Players (Stratified by Highest Level of Football Play)
eTable 4. Characteristics of HITSS Participants Who Completed the Paired Associates Learning (PAL) Versus Those Who Did Not
eTable 5. Associations Between RHI Proxies and Cognitive and Neuropsychiatric Outcomes in the Football Cohort (N=3,970)
eTable 6. Subgroup Analyses Comparing Football Players to Controls
eTable 7. Associations Between RHI Proxies and Cognitive/Neuropsychiatric Outcomes (Excluding Youth)
eReferences

This supplemental material has been provided by the authors to give readers additional information about their work.

eMethods 1. Participant recruitment into the HITSS

Participant recruitment into HITSS was performed via the following channels:

1. Digital Advertising

- Meta Advertising (Facebook and Instagram)
- General advertisements: Targeted to men and women aged 40+ who played soccer or tackle football.
- Targeted advertisements: Focused on specific demographics (gender, sex, race) and in some cases, level of play (e.g., youth sports)

2. Organic Media

- Press release at study launch distributed to large-scale media publications
- Social media outreach: Leveraged well-known soccer and football ambassadors to share videos, quotes, and promotional messages.
- Included both general and targeted campaigns. Specific campaigns aimed to recruit female former soccer players and for former youth football players.

3. In-Person Recruitment

- United Soccer Coaches Annual Convention: Hosted an educational session with prominent former players and maintained a trade show booth to distribute recruitment flyers.
- Concussion Legacy Foundation Annual Gala: Distributed recruitment flyers on-site.

4. Organizational Partnerships

- Collaboration with the Concussion Legacy Foundation
    - Outreach through their newsletter.
    - Inclusion in their Research Registry communications.
- Collaboration with the UCSF Brain Health Registry
    - Broad study outreach via UCSF Brain Health Registry newsletters.

eMethods 2. Assessment of Football History

Participants were first asked the following question: “Have you ever participated in organized sports, which includes membership on a team with scheduled practices and games (excluding pick-up or neighborhood games)?” If yes, they were asked subsequent questions pertaining to sport participation history. For football, participants were asked the following: “Did you play organized American tackle football, which includes membership on a team with scheduled practices and games?” If yes, the participant answered additional questions about their football playing history.

eMethods 3. Description of Study Outcome Measures

Cognitive Outcomes

Cambridge Automated Neuropsychological Battery Paired Associates Learning Test (CANTAB PAL)

CANTAB PAL is a computerized cognitive test assessing episodic memory and visual learning.[1,2,3,4] We examined two PAL outcomes: PAL First Attempt Memory Score (PALFAMS; the number of times a participant chose the correct box on their first attempt when recalling pattern locations), and PAL Total Errors Adjusted (PALTEA; the number of times the participant chose the incorrect box for a stimulus, plus an adjustment for the estimated number of errors they would have made on any problems, attempts, and recalls).

Everyday Cognition Scale (ECog)

The ECog is a 39-item scale measuring subjective changes in cognition (across several domains) and instrumental activities of daily living compared to ten years before.[5] Scores are calculated by averaging the 39 items, and range from 1-4, with higher scores indicating increased subjective cognitive complaints. Items for which a participant selects "I don't know" were coded as missing and excluded from score calculation. An online adaptation of the ECog is administered in both the HITSS and BHR batteries.[6,7]

Behavior Rating Inventory of Executive Function - Adult (BRIEF-A) Meta-Cognition Index (MI)

The meta-cognition index (MI) is a subscale of the BRIEF-A. The BRIEF-A is a standardized, 75-item measure that elicits self-reported information pertaining to executive functioning and self-regulation[38]. The meta-cognition index (MI), a 40-item subscale of the BRIEF-A, assessing measures planning, organizational, and goal-oriented behaviors. We examined MI raw and T-scores for the substudy restricted to the HITSS cohort.

Neuropsychiatric Outcomes

Geriatric Depression Scale -15-item (GDS-15)

The GDS-15 is a self-report 15-item measure that evaluates the presence and severity of depressive symptoms in adults.[8,9]. Higher scores indicate greater severity of depressive symptoms. This analysis included the total score from the GDS-15. BHR and HITSS participants completed GDS-15.

BRIEF-A Behavioral Regulation Index (BRI)

The behavioral regulation index (BRI) is a subscale of the BRIEF-A derived from 30 items assessing an individual's ability to monitor their behavior and regulate impulses.[10] BRI raw and T-scores were examined in HITSS only.

eMethods 4. Collection of Race and Ethnicity in HITSS and BHR

In HITSS and BHR, race is self-reported, and participants may endorse the following options: African American, Asian, Caucasian, Native American, Pacific Islander, Other, Declined to State) Ethnicity is self-reported using the following categories: Latino, Not Latino, Declined to state). In the present analysis, a 3-level race measure was used due to few participants endorsing races other than African American or Caucasian. The “Other” was comprised of Asian, Native American, Pacific Islander, Other, and individuals who endorsed two or more race categories (‘Multiple”).

eMethods 5. Detailed Description of and Rationale for Propensity Score Matching Procedure

Overview

A grid search procedure identified the matching model, and the final model used nearest neighbor matching with propensity scores estimated via logistic regression. We employed a one-to-one matching ratio with replacement, allowing reuse of controls up to three times. The model with the best balance was used. The average standardized mean differences between samples for all variables was less than 0.1, apart from age, which was -0.12

Rationale

We employed 1:1 nearest-neighbor propensity score matching with replacement to prioritize match quality in the presence of limited common support. When overlap between treated and control propensity score distributions is imperfect, matching without replacement can force some treated individuals, especially those matched later in the algorithm, to accept progressively poorer matches because the best controls are removed from the pool. Allowing replacement enables each treated individual to be matched to the closest available control, which typically improves covariate balance and reduces bias, at the cost of modestly higher variance. This bias–variance tradeoff and the rationale for replacement in settings with limited overlap are well described in the matching literature.[11]

Detailed Approach to Matching:

We identified the optimal matching specification using grid search approach. The optimal matching model specification was defined as the one that retained the greatest number of controls matched and achieved best group level balance. Group level balance was assessed using both the maximum absolute standardized mean difference across matching covariates and the standardized mean difference for age specifically. The best model would have the minimum absolute standardized mean difference between groups. For the grid search, we tried all possible combinations of nearest neighbor matching with and without replacement, different ways of estimating propensity scores, different matching ratios, limitations on the number of times a control unit can be re-used. The propensity score estimation methods were logistic regression (glm), generalized additive models (gam), generalized boosted models (gbm), recursive partitioning and regression trees (rpart), and random forest. The matching ratio was varied from 1:1 to 1:3 (football-to-control). The number of times a control unit can be re-used (reuse.max) varied from 1 (no replacement) to, 2 through 10 and to test out whether setting a very large number was needed we also used 1000A caliper was not used for age.

Of the 282 unique controls 70 controls were reused once, 45 were reused twice and 167 were reused thrice. The outcome model handles repeated controls when estimating the standard error.

eFigure 1. Propensity Score Matching Diagnostics

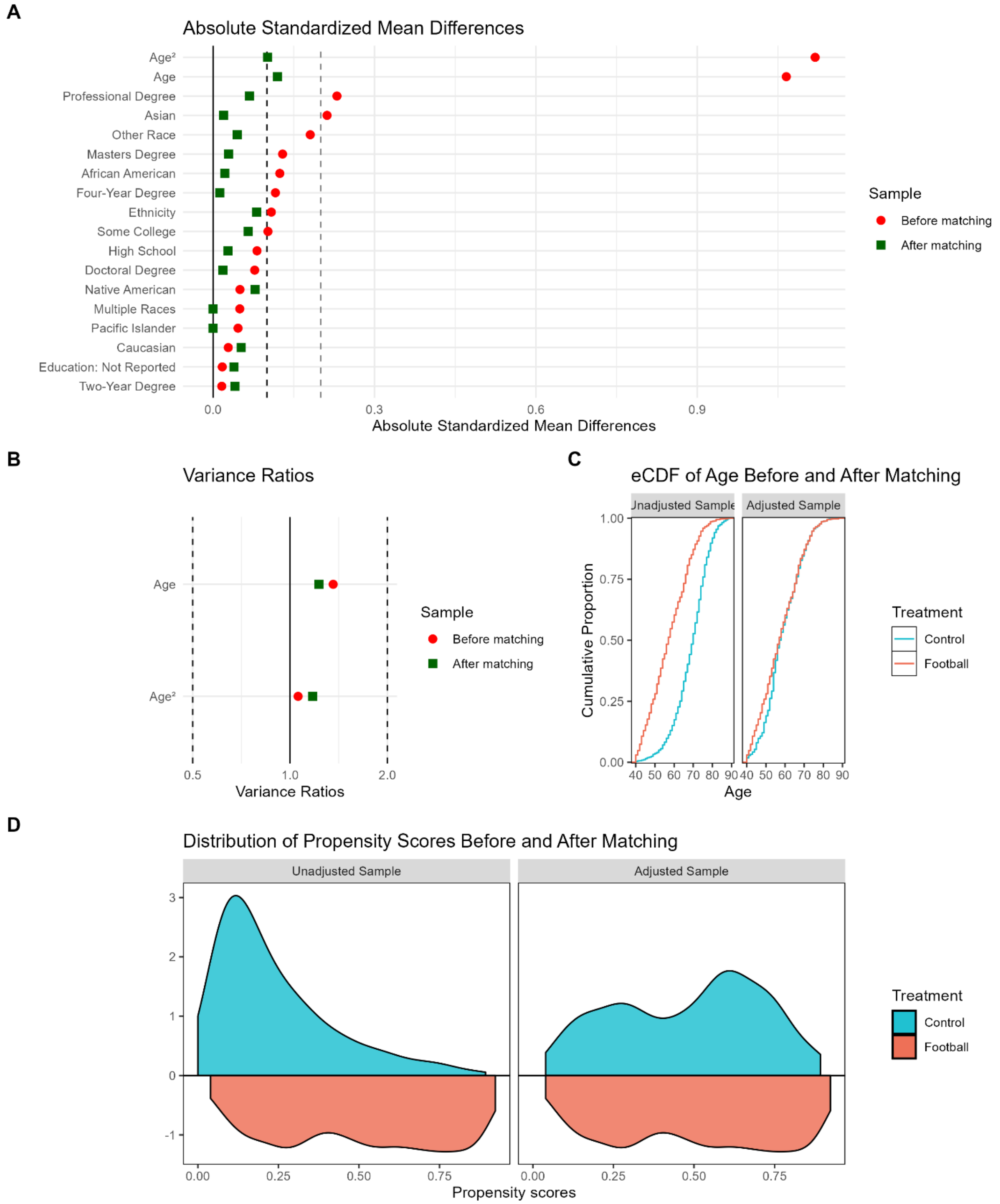

Standardized mean differences (Panel A) and variance ratios (Panel B), with reference lines indicating balance thresholds. Improved balance is shown by green points falling within acceptable thresholds. Panel C shows the empirical cumulative distribution function for age. Panel D displays propensity score distributions.

eFigure 2. Cognitive and Neuropsychiatric Outcomes by Highest Level of Football Play

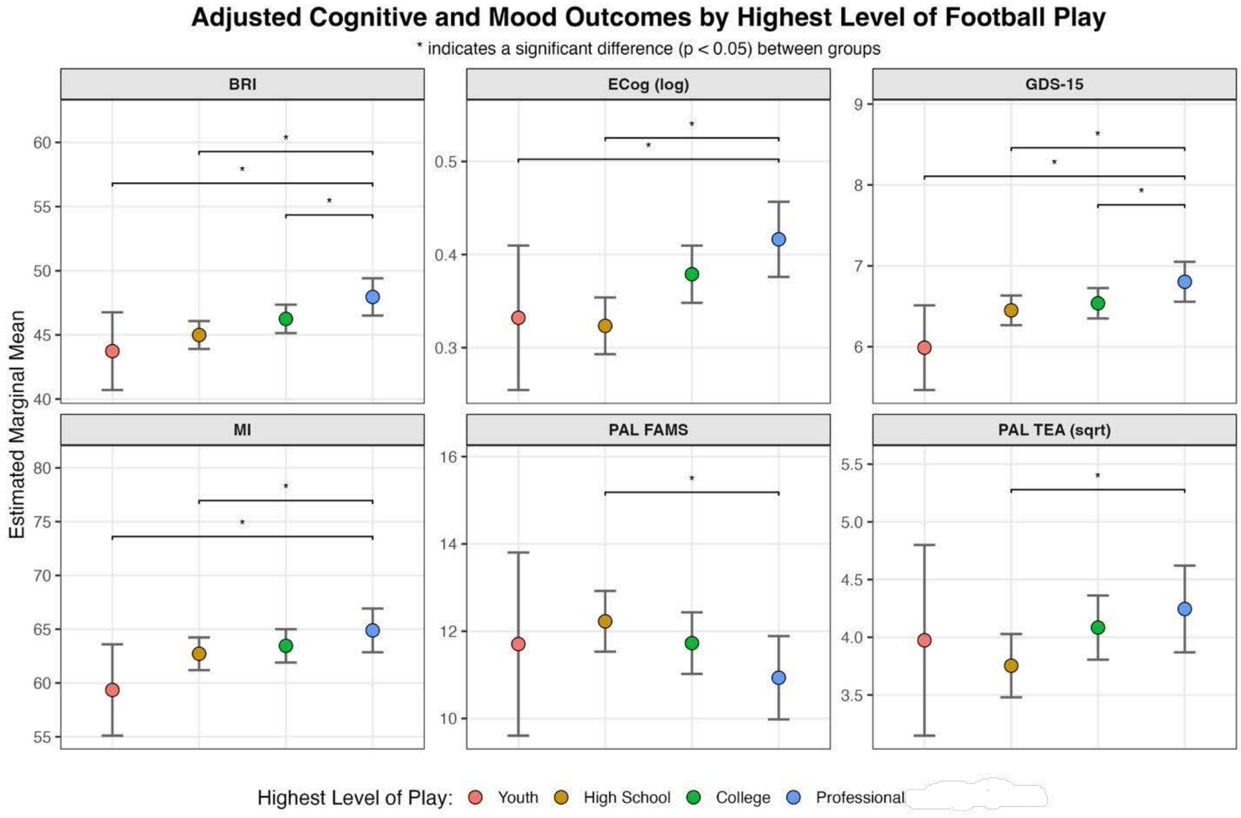

Plots depicting highest level of football play on the x-axis and estimated marginal means of outcome measures on the y-axis. Level of play is separated into professional, college, high school and youth. Estimates are adjusted for age, education, race, and vascular risk. Asterisks indicate a significant difference ($p < 0.05$). ECog was log transformed in analyses and PALTEA was square root transformed due to non-normal distribution of residuals. Higher ECog, BRI, and PALTEA scores indicate worse performance.

eTable 1. Sources of Potential RHI Exposure Besides Football

**Exposure Source**[a]	**N**	**Overall**	**Youth/High School**	**College**	**Pro**	***P* Value**[b]
Soccer	3,841	855 (22%)	470 (26%)	287 (19%)	98 (18%)	<0.001
Ice Hockey	3,813	266 (7.0%)	124 (7.0%)	98 (6.5%)	44 (8.1%)	0.48
Boxing	3,794	203 (5.4%)	88 (5.0%)	62 (4.2%)	53 (9.8%)	<0.001
Futsal	3,797	23 (0.6%)	12 (0.7%)	7 (0.5%)	4 (0.7%)	0.63
Military History	2,426	291 (12%)	185 (16%)	70 (7.2%)	36 (12%)	<0.001
Military Combat	284	72 (25%)	42 (23%)	15 (22%)	15 (43%)	0.04

[a]n (%)

[b]Pearson's Chi-squared test; Fisher's exact test

eTable 2. Outcome Measure Estimated Marginal Means Across Highest Levels of Football Play

Outcome Measure	Highest Level of Play	Adjusted Mean	Standard Error (SE)	95% Confidence Interval (CI)
PAL FAMS	Professional	10.9	0.49	9.98, 11.9
	College	11.7	0.36	11.02, 12.4
	Youth/High School	12.2	0.35	11.52, 12.9
PAL TEA (sqrt)	Professional	4.24	0.19	3.87, 4.62
	College	4.08	0.14	3.81, 4.36
	Youth/High School	3.76	0.14	3.49, 4.03
ECog (log)	Professional	0.42	0.02	0.38, 0.46
	College	0.38	0.02	0.35, 0.41
	Youth/High School	0.32	0.02	0.29, 0.35
GDS-15	Professional	6.80	0.13	6.55, 7.05
	College	6.53	0.10	6.35, 6.72
	Youth/High School	6.42	0.09	6.24, 6.61
BRI	Professional	47.9	0.74	46.5, 49.4
	College	46.2	0.56	45.1, 47.3
	Youth/High School	44.9	0.55	43.8, 46.0
MI	Professional	64.9	1.04	62.8, 66.9
	College	63.4	0.79	61.9, 65.0
	Youth/High School	62.5	0.76	61.0, 64.0

Note: All means are adjusted for race, vascular risk, age, and education

Abbreviations: GDS-15 = Geriatric Depression Scale-15, ECog = Everyday Cognition Scale, PAL FAMS = Paired Associates Learning First Attempt Memory Score, PAL TEA = Paired Associates Learning Total Errors Adjusted, BRIEF = Behavioral Rating Inventory of Executive Function – Adult, MI = Meta Cognition Index, BRI = Behavioral Regulation Index

eTable 3. Characteristics of Controls Versus Football Players (Stratified by Highest Level of Football Play)

Characteristic	Control (N=282)	Youth/HS (N=318)	College (N=275)	Professional (N=68)
**Age**	60.44 (9.71)	56.79 (10.54)	58.31 (10.24)	58.72 (11.47)
**Education Level**				
**High School**	2 (0.7%)	8 (2.5%)	0 (0%)	0 (0%)
**Some College**	22 (7.8%)	40 (13%)	9 (3.3%)	7 (10%)
**2-Year Degree**	17 (6.0%)	27 (8.5%)	4 (1.5%)	4 (5.9%)
**4-Year Degree**	93 (33%)	100 (31%)	114 (41%)	33 (49%)
**Master's Degree**	89 (32%)	98 (31%)	111 (40%)	19 (28%)
**Doctoral Degree**	23 (8.2%)	23 (7.2%)	18 (6.5%)	2 (2.9%)
**Professional Degree**	35 (12%)	21 (6.6%)	19 (6.9%)	3 (4.4%)
**Prefer not to say**	1 (0.4%)	1 (0.3%)	0 (0%)	0 (0%)
**Race**				
**African American/Black**	12 (4.3%)	8 (2.5%)	8 (2.9%)	14 (21%)
**Asian**	3 (1.1)	1 (0.3)	3 (1.1)	0
**Native American**	0 (0%)	1 (0.3%)	3 (1.1%)	0 (0%)
**White**	258 (91%)	291 (92%)	257 (93%)	52 (76%)
**Other Race**	1 (0.4%)	3 (0.9%)	0 (0%)	0 (0%)
**Multiple**	8 (2.8%)	14 (4.4%)	4 (1.5%)	2 (2.9%)
**Ethnicity**				
**Latino**	8 (2.8%)	18 (5.7%)	5 (1.8%)	1 (1.5%)
**Not Latino**	274 (97%)	300 (94%)	270 (98%)	67 (99%)
**PAL FAMS**	12.41 (4.32)	12.76 (4.13)	11.86 (4.17)	10.96 (4.26)
**PAL TEA**	16.06 (14.48)	14.52 (12.63)	18.18 (14.57)	19.99 (15.24)
**ECog**	1.38 (0.43)	1.53 (0.51)	1.56 (0.56)	1.69 (0.69)
**GDS-15**	5.56 (1.48)	6.12 (1.84)	6.23 (1.64)	6.69 (1.85)

Abbreviations: GDS-15 = Geriatric Depression Scale-15, ECog = Everyday Cognition Scale, PAL FAMS = Paired Associates Learning First Attempt Memory Score, PAL TEA = Paired Associates Learning Total Errors Adjusted

eTable 4. Characteristics of HITSS Participants Who Completed the Paired Associates Learning (PAL) Versus Those Who Did Not

Characteristic[a]	Overall N	Completer (N=785)	Non-Completer (N=3,185)	*P* Value[b]
**Age**	3,970	57.74 (10.60)	55.49 (9.80)	<0.001
**Years of Education**	3,961	16.80 (1.98)	15.94 (2.22)	<0.001
**Race**	3,924			
**African American/Black**		36 (4.6%)	435 (14%)	<0.001
**Asian**		4 (0.5%)	12 (0.4%)	
**Native American**		4 (0.5%)	26 (0.8%)	
**Pacific Islander**		0 (0%)	20 (0.6%)	
**White**		704 (90%)	2,494 (79%)	
**Other Race**		6 (0.8%)	61 (1.9%)	
**Multiple**		26 (3.3%)	96 (3.1%)	
**Ethnicity**	3,867			0.11
**Latino**		25 (3.2%)	140 (4.5%)	
**Not Latino**		747 (97%)	2,955 (95%)	
**Years of Play**	3,944	8.37 (4.14)	8.85 (4.72)	0.07
**Level of Play**	3,970			<0.001
**Professional**		83 (11%)	512 (16%)	
**College**		335 (43%)	1,207 (38%)	
**HS/Youth**		367 (47%)	1,466 (46%)	
**AFE**	3,950	11.12 (2.63)	10.64 (2.75)	<0.001
**Linemen (yes)**	3,878	354 (46%)	1,297 (42%)	0.06
**Vascular Risk (Yes)**	2,923	528 (68%)	1,446 (67%)	0.82

[a]Mean (SD); n (%)

[b]Wilcoxon rank sum test; Pearson's Chi-squared test

eTable 5. Associations Between RHI Proxies and Cognitive and Neuropsychiatric Outcomes in the Football Cohort (N=3,970)

Outcome	Factors	Odds Ratio (95% confidence interval)	*P* Value
Ecog	Level of Play[a]		
	Professional	1.36 (1.04, 1.78)	0.02
	College	1.29 (1.08, 1.54)	0.006
	Years of Play	1.03 (1.01, 1.05)	0.003
GDS-15	Level of Play		
	Professional	2.21 (1.32, 3.94)	0.004
	College	1.42 (1.05 1.93)	0.02
	Years of Play	1.07 (1.03, 1.11)	<0.001
BRI	Level of Play		
	Professional	1.61 (1.22, 2.13)	<0.001
	College	1.29, (1.05, 1.58)	0.01
	Years of Play	1.04 (1.02, 1.06)	<0.001

[a]Reference level = Youth/High School

eTable 6. Subgroup Analyses Comparing Football Players to Controls

Results of additional linear regression models with multiway cluster-robust standard errors comparing football players across different levels of play (youth/high school, college, professional) to non-RHI exposed controls enrolled in the UCSF Brain Health Registry. Controls are used as the reference group in all models. Models adjusted for age.

Outcome	Factors	B (95% confidence interval)	P-Value
**PAL FAMS**	Youth/High School	-0.20(-0.86, 0.45)	0.54
	College	-0.87 (-1.55, -0.19)	**0.01**
	Professional	-1.71(-2.79, -0.63)	**0.002**
	Age	-0.15(-0.18, -0.12)	**<0.001**
**PAL TEA (Square Root)**	Youth/High School	0.09(-0.17, 0.35)	0.49
	College	0.47(0.20, 0.73)	**<0.001**
	Professional	0.65(0.22, 1.09)	**0.003**
	Age	0.07(0.06, 0.08)	**<0.001**
**ECOG (Log)**	Youth/High School	0.10(0.05, 0.14)	**<0.001**
	College	0.11(0.06, 0.16)	**<0.001**
	Professional	0.17(0.08, 0.26)	**<0.001**
	Age	-0.0003(-0.002, 0.002)	0.73
**GDS-15**	Youth/High School	0.50(0.22, 0.78)	**<0.001**
	College	0.64(0.37, 0.91)	**<0.001**
	Professional	1.11(0.64, 1.58)	**<0.001**
	Age	-0.02(-0.03, -0.009)	**<0.001**

eTable 7. Associations Between RHI Proxies and Cognitive/Neuropsychiatric Outcomes (Excluding Youth)

Outcome	Factors	B (95% confidence interval) or F-statistic	Adjusted P-Value
**PAL FAMS**	AFE	0.002(-0.12, 0.12)	0.98
	Years of Play	-0.06(-0.13, 0.002)	0.06
	Linemen (yes)	0.09(-0.47, 0.64)	0.76
	Level of Play	3.95	**0.03**
**PAL TEA (Square Root)**	AFE	-0.003(-0.05, 0.04)	0.98
	Years of Play	0.03(0.006, 0.06)	**0.04**
	Linemen (yes)	-0.04(-0.26, 0.18)	0.76
	Level of Play	5.60	**0.008**
**ECOG (Log)**	AFE	-0.003(-0.009, 0.002)	0.40
	Years of Play	0.006(0.003, 0.009)	**<0.001**
	Linemen (yes)	-0.02(-0.05, 0.002)	0.29
	Level of Play	11.63	**<0.001**
**GDS**	AFE	0.007(-0.03, 0.04)	0.69
	Years of Play	0.02(0.005, 0.04)	**0.01**
	Linemen (yes)	0.03(-0.13, 0.19)	0.71

	Level of Play	3.87	**0.02**
**BRI**	AFE	-0.14(-0.34, 0.07)	0.37
	Years of Play	0.26(0.15, 0.36)	**<0.001**
	Linemen (yes)	0.18(-0.78, 1.15)	0.71
	Level of Play	8.16	**<0.001**
**MI**	AFE	-0.20(-0.48, 0.09)	0.40
	Years of Play	0.15(-0.003, 0.30)	0.06
	Linemen (yes)	0.58(-0.78, 1.93)	0.76
	Level of Play	2.06	0.13

Abbreviations: AFE = age of first exposure to football, GDS-15 = Geriatric Depression Scale-15, ECog = Everyday Cognition Scale, PAL FAMS = Paired Associates Learning First Attempt Memory Score, PAL TEA = Paired Associates Learning Total Errors Adjusted, BRIEF = Behavioral Rating Inventory of Executive Function – Adult, MI = Meta Cognition Index, BRI = Behavioral Regulation Index

eReferences

1. Barnett JH, Blackwell AD, Sahakian BJ, Robbins TW. The Paired Associates Learning (PAL) Test: 30 Years of CANTAB Translational Neuroscience from Laboratory to Bedside in Dementia Research. *Curr Top Behav Neurosci*. 2016;28:449-474. doi:10.1007/7854_2015_5001

2. Junkkila J, Oja S, Laine M, Karrasch M. Applicability of the CANTAB-PAL computerized memory test in identifying amnestic mild cognitive impairment and Alzheimer's disease. *Dement Geriatr Cogn Disord*. 2012;34(2):83-89. doi:10.1159/000342116

3. Mitchell J, Arnold R, Dawson K, Nestor PJ, Hodges JR. Outcome in subgroups of mild cognitive impairment (MCI) is highly predictable using a simple algorithm. *J Neurol*. 2009;256(9):1500-1509. doi:10.1007/s00415-009-5152-0

4. Ashford MT, Aaronson A, Kwang W, et al. Unsupervised Online Paired Associates Learning Task from the Cambridge Neuropsychological Test Automated Battery (CANTAB®) in the Brain Health Registry. *J Prev Alzheimers Dis*. 2024;11(2):514-524. doi:10.14283/jpad.2023.117

5. Farias ST, Mungas D, Reed BR, et al. The measurement of everyday cognition (ECog): scale development and psychometric properties. *Neuropsychology*. 2008;22(4):531-544. doi:10.1037/0894-4105.22.4.531

6. Nosheny RL, Camacho MR, Insel PS, et al. Online study partner-reported cognitive decline in the Brain Health Registry. *Alzheimers Dement N Y N*. 2018;4:565-574. doi:10.1016/j.trci.2018.09.008

7. Aaronson A, Ashford MT, Jin C, et al. Brain Health Registry Study Partner Portal: Novel infrastructure for digital, dyadic data collection. *Alzheimers Dement*. 2024;20(2):846-857. doi:10.1002/alz.13492

8. Yesavage JA, Brink TL, Rose TL, et al. Development and validation of a geriatric depression screening scale: a preliminary report. *J Psychiatr Res*. 1982;17(1):37-49. doi:10.1016/0022-3956(82)90033-4

9. Guerin JM, Copersino ML, Schretlen DJ. Clinical utility of the 15-item geriatric depression scale (GDS-15) for use with young and middle-aged adults. *J Affect Disord*. 2018;241:59-62. doi:10.1016/j.jad.2018.07.038

10. Gioia GA, Isquith PK, Guy SC, Kenworthy L. TEST REVIEW Behavior Rating Inventory of Executive Function. *Child Neuropsychol*. Published online September 1, 2000. doi:10.1076/chin.6.3.235.3152

11. Stuart EA. Matching methods for causal inference: A review and a look forward. *Stat Sci Rev J Inst Math Stat*. 2010;25(1):1-21. doi:10.1214/09-STS313
