# Supplementary material for: Cognitive and Neuropsychiatric Function in Former American Football Players
Source: JAMA Netw Open. 2026 Feb 27;9(2):e2560077. doi: 10.1001/jamanetworkopen.2025.60077 (PMC12949441; doi:10.1001/jamanetworkopen.2025.60077)
Supplement: Supplement 2. — Data Sharing Statement [file jamanetwopen-e2560077-s002.pdf]

Data Sharing Statement

Aaronson. Cognitive and Neuropsychiatric Function in Former American Football Players. *JAMA Netw Open*. Published February 27, 2026. doi:10.1001/jamanetworkopen.2025.60077

Data
Data available: Yes
Data types: Deidentified participant data
How to access data: Data will be able to be requested from FITBIR: https://fitbir.nih.gov/content/access-data
When available: With publication

Supporting Documents
Document types: None

Additional Information
Who can access the data: Researchers whose proposed use of the data has been approved
Types of analyses: N/A
Mechanisms of data availability: After approval by FITBIR
